# The Bacterial Spore as a Mucosal Vaccine Delivery System

**DOI:** 10.3390/ijms241310880

**Published:** 2023-06-29

**Authors:** Anella Saggese, Loredana Baccigalupi, Giuliana Donadio, Ezio Ricca, Rachele Isticato

**Affiliations:** 1 Department of Biology, Federico II University, 80126 Naples, Italy; anella.saggese@unina.it (A.S.); isticato@unina.it (R.I.); 2 Department of Molecular Medicine and Medical Biotechnology, Federico II University, 80131 Naples, Italy; lorbacci@unina.it; 3 Department of Pharmacy, University of Salerno, 84084 Salerno, Italy; gdonadio@unisa.it

**Keywords:** spores, mucosal vaccines, vaccine delivery, *Bacillus subtilis*, spore surface display

## Abstract

The development of efficient mucosal vaccines is strongly dependent on the use of appropriate vectors. Various biological systems or synthetic nanoparticles have been proposed to display and deliver antigens to mucosal surfaces. The *Bacillus* spore, a metabolically quiescent and extremely resistant cell, has also been proposed as a mucosal vaccine delivery system and shown able to conjugate the advantages of live and synthetic systems. Several antigens have been displayed on the spore by either recombinant or non-recombinant approaches, and antigen-specific immune responses have been observed in animals immunized by the oral or nasal route. Here we review the use of the bacterial spore as a mucosal vaccine vehicle focusing on the advantages and drawbacks of using the spore and of the recombinant vs. non-recombinant approach to display antigens on the spore surface. An overview of the immune responses induced by antigen-displaying spores so far tested in animals is presented and discussed.

## 1. Introduction

Vaccinations are the most effective strategy to control bacterial and viral infections. The World Health Organization (WHO) considers immunization campaigns as global health success stories, estimating that they prevent millions of deaths yearly (https://www.who.int/health-topics/vaccines-and-immunization (accessed on 14 April 2023)). By mimicking the pathogen infection, vaccines induce the activation of the immune system and, therefore, the response against the pathogen [1]. After the immunization, the delivered antigen is recognized by the pattern recognition receptors (PRRs) of the innate immune cells (macrophages and dendritic cells), stimulating the production of cytokines and chemokines and leading to an increase in the number of antigen-presenting cells (APCs). These uptake, process, and present the antigen to the T cells that, in turn, induce B cells to produce antigen-specific antibodies [1,2]. When the pathogen has been eliminated, the adaptative immune system develops the immunological memory, the basis of long-term protection and the final goal of a vaccine, since it leads to the persistence of antibodies and the generation of memory cells able to quickly react upon re-exposure to the same pathogen [1,2].

Vaccines can be administered through either parenteral or mucosal routes. Most authorized vaccines are administered parenterally, i.e., injected subcutaneously (SC), intradermally (ID), or intramuscularly (IM). All three parenteral routes of vaccine administration have advantages and pitfalls, with the ID route inducing a stronger immune response than IM or SC, but also requiring special devices for the administration and causing more serious adverse reactions at the administration site [3]. In general, parenteral vaccines elicit a strong immune response but weak mucosal protection, and do not prevent infection by the pathogen [4]. In addition, they require trained personnel for the administration (injection), making immunization campaigns difficult and expensive, especially in third-world countries [3]. On the other hand, mucosal vaccines are expected to induce protective cellular and humoral responses at both mucosal and systemic levels [5,6]. A strong induction of adaptive immunity at mucosal sites, involving secretory antibodies and tissue-resident T cells, would prevent pathogen entry into the animal body, thus preventing the infection by the pathogen and its transmission [7]. Since mucosal vaccines do not require an injection, they are not invasive and are easy to administrate facilitating mass immunization campaigns. The use of oral vaccinations is also preferable for animal husbandry and aquaculture, since they reduce the costs of animal management and the stress caused to animals [8]. However, mucosal vaccines also have drawbacks that have so far limited their use. Oral vaccines generally have low immunogenicity due to the high levels of tolerance induced by ingested antigens and to the lack of efficient mucosal adjuvants and delivery systems able to prevent antigen degradation at the mucosal sites [7]. Therefore, antigens administrated by the mucosal route often cannot efficiently reach the inductive site of the mucosa-associated lymphoid tissues (see below) and trigger a strong immune response. As a consequence, the effectiveness of mucosal vaccines is often hindered and only a few mucosal vaccines, all based on live attenuated or death-inactivated pathogens, are currently approved for human use (Table 1).

This review will discuss the recent advancements in mucosal delivery systems, focusing on the use of bacterial spores as vaccine vehicles able to conjugate the advantages of live cells and synthetic nanoparticles.

## 2. Mucosal Surfaces and Mucosal Immune System

**Figure 1:**
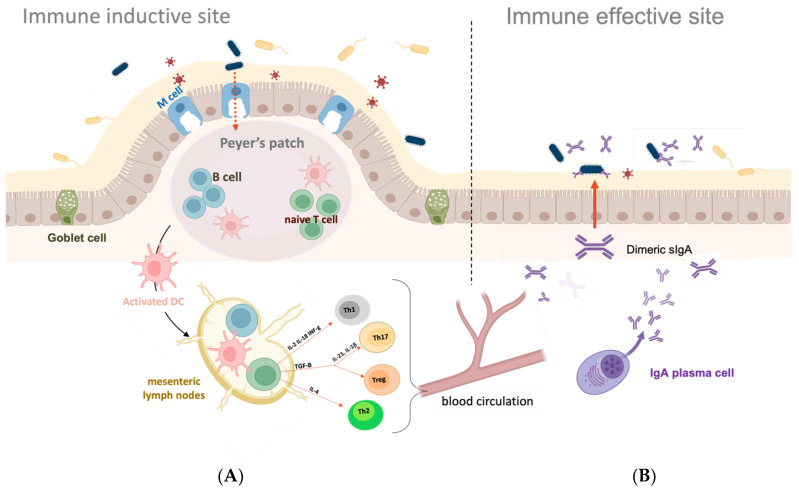
Gut-associated mucosal immune system. (**A**) Antigens within the intestinal lumen are transferred to the Peyer’s patches via M cells and recognized by DCs. In the mesenteric lymph nodes, the activated DCs prime naïve T cells by the secretion of tissue-specific adhesion molecules and cytokines. The T helper cells are responsible for fighting different types of pathogens. Th1s secrete IFN-γ, activating macrophages and cytotoxic T cells against intracellular pathogens. Th2 cells produce IL-4, IL-5, and IL-13, which activate the humoral immune responses against extracellular pathogens by the activation of B cells. Finally, Th17s are responsible for antifungal and antibacterial immunity. T cell-dependent activation of IgA-committed B cells is also induced. (**B**) Then, antigen-specific T cells and IgA-committed B cells migrate to effector sites through blood circulation. IgA-committed B cells differentiate into IgA-producing plasma cells in the presence of cytokines produced by Th2 cells, and they subsequently produce dimeric forms of IgA. Finally, the IgA dimeric forms are secreted and released in the intestinal tract, where they play critical roles in mucosal immune responses, such as immune exclusion, antigen excretion, and intracellular virus neutralization. Created with https://www.biorender.com/ (accessed on 2 May 2023).

The mucosal surfaces of the human body present structural and functional differences at the various body sites, i.e., the gastrointestinal, urogenital, respiratory tracts and oral and ocular cavities. In the gastrointestinal tract (GIT), the mucus is secreted by the Goblet cells and covers columnar epithelial cells. Antigens are transported from the lumen to dendritic cells (DCs) mainly through M cells, allowing antigen presentation to the mucosal immune system, and inducing the production of IgG and secretory IgA (sIgA) (Figure 1). A similar structure with Goblet and M cells interspersed with columnar epithelial cells is observed in the nasal cavity, where a fibrous layer with lymphocite-enriched zones is beneath the epithelium. In other cases, such as the oral and ocular cavities, the mucus layer covers a multilayer squamous epithelium and no M cells and Goblet cells are present. In these cases, other tissues (glands) secrete the mucus, the DCs migrate to the adjacent lymph nodes upon antigen recognition, and only an IgG response is induced without the production of sIgA [9].

The mucosal surfaces represent contact sites between the body and the external environment and are in direct contact with all microbes, viruses, and molecules present in air, water, and foods. Physical, chemical, and immunological barriers are then essential to protect the mucosal surfaces and maintain homeostasis, avoiding chronic inflammatory responses due to the high antigen load. The dense mucus layer, which prevents adherence to the epithelium, and the tight junctions, connecting epithelial cells and controlling access to the underlying tissues, are the physical barriers protecting the mucosal surfaces. Such physical protection is aided by pH differences and antimicrobial substances (biochemical barriers) and by the action of the innate and adaptive immune systems (immunological barriers) [2]. The innate immune system is naturally present in the organism and is the first line of defense to respond quickly to bacteria and antigens. It recognizes a limited number of evolutionarily conserved molecules and does not retain a memory of a previous response. On the other hand, the adaptive immune system is acquired during lifetime upon the exposure to pathogens, is highly specific and retains a memory of a previous response. Both systems cooperate to efficiently recognize and eliminate pathogens. The immune system is, in part, located along the mucosal surfaces (mucosal immune system) and has an important role in immune surveillance.

The mucosal immune response is coordinated by the mucosa-associated lymphoid tissue (MALT) diffused in various submucosal sites. The MALT is composed of DC, macrophages, intraepithelial T cells (CD8+), regulatory T cells (Treg), and plasma cells that are organized into inductive sites, where antigens are recognized, and effector sites where the adaptive immune responses are mediated (Figure 1) [10,11]. The MALT is commonly subdivided into mucosa-specific lymphoid tissues, for example, the gut-associated lymphoid tissue (GALT) or the nasal-associated lymphoid tissue (NALT). In the intestine, a large number of immune cells are found beneath the Peyer’s patches (PP) that are formed by groups of M cells (specialized phagocytic cells with high transcytotic activity) [11]. At the level of the PP, antigens are transported from the intestinal lumen across the intestinal barrier and are taken up by the DCs and presented to naïve T cells in the local mesenteric lymph nodes [12]. In this district, the activated DCs promote naïve T cell differentiation into distinct T helper cells (Th1, Th2, or Th17) or T reg cells by secreting different types of pro-inflammatory cytokines (Figure 1A). In a health situation, DCs produce IL-10 and TGF-β inducing tolerance; thus, DCs have a dual function: they either boost the immune system or dampen it, leading to tolerance and maintenance of the immune homeostasis. In a stimulus-dependent manner, IL-2, IL-18, and INF-γ induce a Th1 response, IL-4 induces a Th2 response, and IL-6 and IL-23 induce a Th17 response [10]. In turn, the T cells interact with B cells to promote antibody production at multiple mucosal sites. In particular, activated B cells produce antigen-specific IgG and secretory IgA (sIgA) antibodies [10,11].

Although the mucosal tissues of the body share some common features, each tissue has a peculiar structure (epithelia, mucus, lymphoid structures, and resident immune cells) and a specific commensal microbiota. These specificities affect the nature of the immune inductive sites (GALT and mesenteric lymph nodes in the intestine, NALT and Cervical lymph nodes in the nose), the type of immune response, and its duration. Therefore, all these factors affect the mucosal immune response and are relevant for the design of a mucosal vaccine. The various mucosal sites induce different immune responses with oral vaccinations that are particularly adapted to induce a response in the GIT, salivary glands, and mammary glands, and nasal vaccinations that are particularly adapted for an immune response in respiratory, gastric, and genital tracts [13]. However, the various mucosae are connected and, therefore, immunization at a single site promotes at least some immune responses at distant sites [14,15]. A full understanding of the crosstalk between the various mucosal sites is critical to designing novel vaccines that can potentially target mucosae distant from the vaccination site.

## 3. Mucosal Adjuvants and Delivery Systems

A full development of mucosal vaccines has been so far limited by the lack of appropriate antigens, adjuvants, and delivery systems. Most antigens, when exposed to the harsh environmental conditions of the mucosa, are degraded before they can be recognized by mucosal APCs (including DCs, macrophages, B cells) [16]. Although typical adjuvants used in parenteral vaccinations, for example, aluminum hydroxide (alum), complete Freund’s adjuvant, and incomplete Freund’s adjuvant, do not successfully stimulate mucosal immune responses, significant progresses have recently been made to develop appropriate mucosal adjuvants.

### 3.1. Mucosal Adjuvants

A vaccine adjuvant is a molecule or particle able to activate innate immunity by inducing the production of proinflammatory molecules, chemokines, and cytokines by APCs. In recent years, various mucosal adjuvants have been proposed and extensively reviewed [9,16]. Adjuvants proposed for mucosal vaccinations include modified bacterial toxins, flagellin, and other immunomodulatory molecules of bacterial or cellular origin [16]. The best characterized mucosal adjuvants are non-toxic variants of bacterial enterotoxins, in particular, of the cholera toxin (CT) of *Vibrio cholerae* and of the heat-labile toxins (LT) of enterotoxigenic strains of *Escherichia coli* (ETEC). The multiple mutant cholera toxin (mmCT) and the double mutant heat-labile toxin (dmLT), carrying, respectively, multiple mutations in the A subunit of the CT or two amino acid replacements (R192G and L211A) in LT, have low or no toxicity, but retain the adjuvant activity of the full toxins and have been shown to potentiate the immune responses of various experimental mucosal vaccines against pathogens, such as *Streptococcus pneumoniae*, *Helicobacter pylori*, *V. cholera*, and ETEC [16,17]. Also, flagellin, the main structural component of the bacterial flagella, has been proposed as a mucosal adjuvant. Orally dosed flagellin of *Salmonella typhimurium* induces an inflammatory response by interacting with the Toll-Like receptor 5 (TLR5) and was shown to potentiate the immune response induced by mucosally administered bacterial and viral antigens [16]. In addition to enterotoxins and flagellin, other molecules have been proposed as mucosal adjuvants, including enterocyte binding proteins (for example, InlA of *Listeria monocytogenes* and FnBPA of *Staphylococcus aureus*) [18], cell surface proteins from the protozoan *Giardia lamblia*, M cell-targeting peptides [19], DC-targeting molecules [20], cytokine-derived molecules, and the Fc region of antibodies [21]. Immune potentiator molecules, such as cytokines, have also been proposed as mucosal adjuvants, particularly for vaccines based on live-attenuated pathogens and/or live vectors (non-pathogenic microorganisms modified to express heterologous antigens) [22]. Interferon-γ (IFN-γ), IL-1β, IL-12, IL-6, IL-10, and IL-12 have all been tested as adjuvants of live-attenuated vaccines against a variety of infectious diseases [22].

Quiescent spores of *Bacillus toyonensis*, a widely used animal probiotic, have been shown to act as a parenteral [23] and mucosal [24] adjuvant of vaccines against a *Clostridium perfringens* infection and the tetanus toxin, respectively.

### 3.2. Mucosal Delivery Systems

Various approaches have been used to deliver antigens to mucosal surfaces, including live cells, bacterial-derived vesicles, viruses, virus-like particles, and synthetic materials. All these techniques rely either on the discovery and optimization of proteins/peptides to be used as antigens (subunit vaccines) or on the delivery of DNA/RNA coding for antigens (genetic vaccines) [25]. For subunit vaccines, antigens can be either produced in vitro and later formulated in vaccine preparations, or their coding genes can be used to obtain antigen synthesis in vivo by a live vaccine carrier. Several pathogenic and non-pathogenic bacteria have been tested as live vehicles of heterologous antigens [26,27]. Initially, epitopes of the hepatitis B virus [28], of the cholera toxin [29], or of the parasite *Plasmodium falciparum* [30] were displayed on the surface of non-virulent strains of the pathogenic bacterium *Salmonella.* Antigen-specific immune responses were observed when the recombinant bacterial cells were used for the mucosal immunization of mice, providing clear evidence that an attenuated bacterium can be effectively used as a vaccine vehicle to deliver heterologous antigens [29,30]. Later on, non-pathogenic, commensal bacteria were also used to display and deliver antigens to mucosal surfaces to avoid the use of engineered, attenuated pathogens [31].

More recently, bacterial-derived materials have been proposed as mucosal vaccine vehicles. In this context, outer membrane vesicles (OMVs), non-living structures deriving from gram-negative bacteria, stimulated particular interest [32]. OMVs are formed during bacterial growth by either a spontaneous or induced budding of the outer membrane. OMVs obtained from gram-negative pathogens contain periplasmic material and molecules normally exposed on the outer membrane, and a parenteral vaccine against *Neisseria meningitidis* based on OMVs has already been licensed [32]. OMVs can also be obtained from non-pathogenic, gram-negative bacteria genetically engineered to express a bacterial or non-bacterial antigen on the cell surface. Recently, a nasally delivered vaccine based on OMVs was shown to induce high mucosal and systemic protection against a SARS-CoV-2 infection [33].

A variety of either natural or synthetic molecules has been proposed to deliver and protect antigens at specific mucosal sites [9,16,25,34]. These include micro- or nano-particles made of polyanhydrides (PHAs), poly (ethylene-glycol), poly (lactic acid), chitin, alginate, and several other polymers that are not cytotoxic, biocompatible, mucoadhesive, and that, by different techniques, are used to coat antigens, therefore protecting them and increasing their immunogenicity [16,34].

The use of liposomes to deliver antigens to the mucosal surfaces has also attracted much interest in the possibility of combining the delivery with the adjuvant effect [35]. Liposomes are micro- or nano-metric lipid bilayers that can contain or expose antigens and be constructed to have an immunomodulatory activity that can induce innate and adaptive responses [35].

Self-assembling protein nanoparticles, such as virus-like particles or nanoparticles displaying antigens, have been shown to facilitate antigen uptake and presentation and are widely applied in developing new vaccines. However, their use has been so far mainly focused on parenteral vaccinations [36,37].

Various viral vectors, including adenovirus, influenza virus, Newcastle disease virus (NDV), and other paramyxoviruses, have been proposed as vaccine vectors, mainly for parenteral immunizations, but more recently for mucosal ones as well [38].

In addition to live bacteria, bacterial or viral-derived particles, and synthetic materials, dead or quiescent cells have also been proposed as mucosal vaccine vectors. An example of dead cells is bacterial ghosts (BGs), empty bacterial envelopes of gram-negative bacteria that do not have nucleic acids. BGs attracted the scientific community’s attention for the possibility of using highly immunogenic pathogens, also genetically engineered, to express additional antigens or adjuvants on their surface without the risk associated with the live cells [39]. BGs differ from classical heat- or chemically-inactivated pathogens that have long been used as vaccines for their preparation methods. Classical methods to produce inactivated vaccines were based on formaldehyde or heat treatments that can damage the surface structures (antigens) and reduce the immunogenicity of the vaccine. BGs can be prepared by genetic (expression of lytic enzymes, phage proteins, or antimicrobial peptides) or by chemical (treatments with NaOH, SDS or H_2_O_2_) approaches, all able to strongly reduce viability without altering the surface structures of the cells [39].

The best-characterized example of quiescent cells used as vaccine delivery systems is the bacterial (endo)spore produced by the gram-positive bacterium *Bacillus subtilis*. A genetically engineered spore expressing the C fragment of the tetanus toxin on its surface [40], induced a protective, mucosal, and systemic immune response [41]. Over the years, several other antigens have been displayed on the spore of *B. subtilis*, as well as on spores of other *Bacillus* species [27,42], and this spore-display system will be discussed in detail in the following paragraphs.

## 4. The *Bacillus* Spore as Mucosal Vaccine Vehicles

### 4.1. The Bacillus Spore

**Figure 2:**
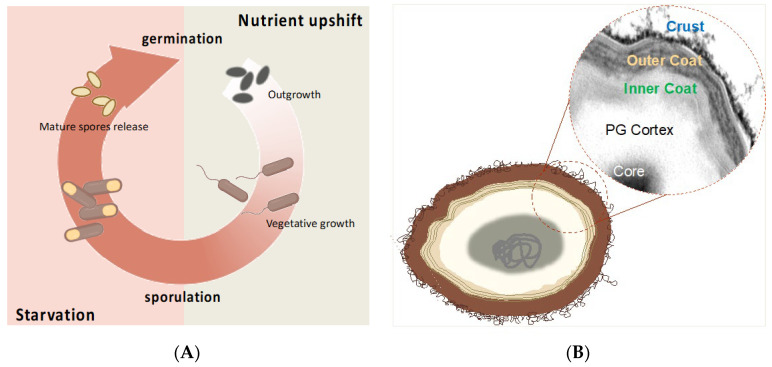
Schematic view of sporulation process (**A**) and spore structure (**B**) of *B. subtilis*. (**A**) In harsh conditions, such as nutrient depletion, the vegetative cells start the alternative cycle of sporulation. Mature released spores can remain dormant for an indefinite period before they germinate and resume the vegetative cycle. Adapted from Mutlu (2018) [44]. (**B**) Cartoon of a typical *B. subtilis* spore. Spore structural layers of the spore protect the genome contained in the partially dehydrated core. The dotted circle shows a detail of TEM micrograph of a spore stained with ruthenium red. Created with https://www.biorender.com/ (accessed on 2 May 2023).

The *B. subtilis* spore, like that produced by most members of the *Bacillus* and *Clostridium* genera, is a particularly stable and resistant cell formed in the cytoplasm of a vegetative cell when the environmental conditions no longer allow cell growth and/or survival (Figure 2A) [43]. The released spore can survive indefinitely in the absence of water and nutrients, and in the presence of toxic chemicals, lytic enzymes, and extremes of temperature and pH [43]. The quiescent spore responds to the renewed presence of nutrients and favorable conditions by germinating and, thus, originating vegetative cells able to grow and eventually sporulate again (Figure 2A) [44].

The spore stability and resistance are in part due to its peculiar structure. A partially dehydrated cytoplasm contains a copy of the chromosome and forms the spore interior (core), which is surrounded and protected by a thick peptidoglycan-like cortex, a multilayered, proteinaceous coat, and, in *B. subtilis*, a crust made of proteins and glycoproteins (Figure 2B). Other spore-forming species either do not have an additional layer outside the coat or have an exosporium, a balloon-like structure also made of glycoproteins that loosely surrounds the spore and mediates its interactions with the environment [45]. Proteins and glycoproteins on the outermost spore layers [46,47] make the spore surface negatively charged and relatively hydrophilic [46,48]. Some spore surface proteins self-assemble around the spore [49,50,51], forming remarkably robust structures [52].

In addition to a survival strategy, producing a spore is also a successful mechanism for the dispersal of these organisms on Earth. Spores are found in almost every environmental niche, including the gut of terrestrial and aquatic animals [53,54]. Seminal experiments with a murine model have shown that ingested spores of *B. subtilis* safely transit the stomach, germinate, and proliferate in the upper part of the intestine [55], and that re-sporulate in the lower part of the intestine [56]. In the GIT, *B. subtilis* spores and germination-derived cells interact with intestinal epithelial and immune cells, contributing to the normal development of the GALT [57,58] and protecting the host from enteropathogens [59]. Also, based on these properties, spores of several *Bacillus* species are widely commercialized as probiotic preparations for animals and humans [60,61].

### 4.2. The Spore Delivery Systems: Recombinant Approach

The spore structure and stability suggested its use as a platform to display heterologous molecules [40]. In a proof-of-concept study, the coat protein CotB of *B. subtilis* was selected as a carrier for the spore display of a model passenger protein, the C fragment of the tetanus toxin (TTFC) of *C. tetani* [40]. DNA coding for TTFC was fused in frame with the *cotB* gene and inserted on the *B. subtilis* chromosome under the transcription and translation signals of *cotB*, thus ensuring genetic stability and proper expression of the chimera [40]. An average of 1.5 × 10^3^ TTFC molecules per spore was displayed [40], and mucosally administered spores were able to induce an antigen-specific immune response in mice [41].

**Figure 3:**
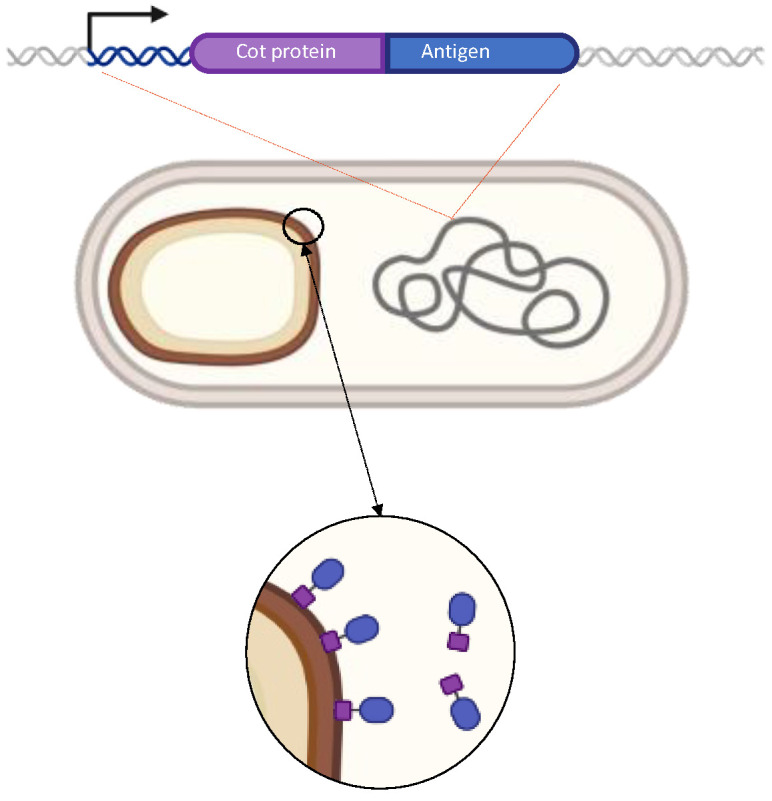
Strategy of recombinant spore-surface display. A gene fusion is constructed between DNA coding for a coat protein (violet) and for an antigen (blue). The fusion is under the transcriptional and translational signals of the spore surface gene and is integrated into *B. subtilis* chromosome. During sporulation, the recombinant protein is expressed in the mother cell and assembled on the forming spore.

The gene fusion, integrated on the chromosome and expressed under the transcription and translation signals of the anchor protein, produces a chimera in the mother cell. The chimera is then driven on the spore surface by the spore surface protein used as an anchor, thus, leading to the release of a mature spore fully decorated with the selected antigen (Figure 3).

The same recombinant approach has been used to display a variety of antigens fused to CotB and several other coat proteins as an anchor (Table 2).

Sets of either replicative [91] or integrative [92] plasmids have been developed to facilitate the construction of gene fusions. The integrative vectors, allowing the integration of the gene fusions on the *B. subtilis* chromosome, grant a better genetic stability over replicative plasmids. Spores of species other than *B. subtilis* have also been considered for displaying heterologous proteins [97,98], but so far, not yet tested for expressing antigens.

Recombinant spores have also been proposed as vaccine vehicles for veterinary uses [68,99], and for aquaculture [89,90,93].

Various alternative recombinant approaches to display proteins on spores have also been proposed. Examples include enzymes [100] and the protective antigen of *B. anthracis* [101] that were over-expressed in the mother cell of sporulating cells of *B. subtilis,* and part of the highly concentrated heterologous proteins spontaneously adsorbed around the forming spores, decorating the released spore. This approach is not based on using a spore surface protein as an anchor, but rather on the spontaneous attachment of highly concentrated proteins on the spore surface. This concept was previously exploited as a non-recombinant spore display [102,103], and is discussed below.

A different example of recombinant spore-display is the reconstitution of the basement layer of the *B. subtilis* coat around spherical membranes supported by silica beads. Such artificial spore-like particles (synthetic spore husk-encased lipid bilayers, SSHELs) were covalently bound to small molecules and suggested as a versatile display platform for drugs, antigens, and enzymes [104].

More recently, antigenic peptides of the zoonotic intestinal tapeworm of dogs *Echinococcus granulosus* have been fused to the TasA protein of *B. subtilis* [105]. TasA is a major component of the extracellular matrix that, by forming amyloid-like fibers, supports the assembly of the biofilm. TasA-fused antigens were orally administered to dogs and shown able to induce an immune response with production of antigen-specific IgG, but not sIgA [106]. Interestingly, an immunohistochemistry analysis strongly suggested that the sera of immunized dogs recognize the infective form of the parasite [106].

### 4.3. The Spore Delivery Systems: Non-Recombinant Approach

**Figure 4:**
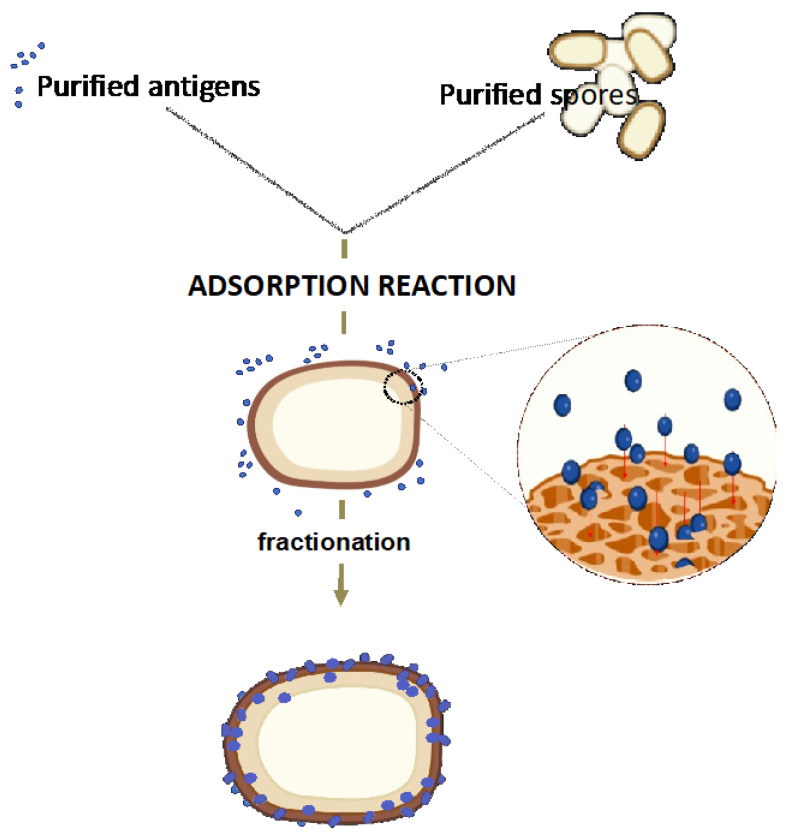
Strategy of non-recombinant spore-surface display. Purified antigens and spores are reacted in an acidic reaction buffer. During the adsorption reaction (1 h), antigens are adsorbed on the spore surface or can infiltrate through the pores formed by spore surface coat proteins.

The non-recombinant spore display system (spore-adsorption) has been recently reviewed [103]. It is based on the spontaneous and stable binding of a purified heterologous protein to the spore surface (Figure 4). This approach has been used to adsorb on the *B. subtilis* spore various enzymes [103] and model antigens, such as TTFC of *C. tetani*, PA of *B. anthracis*, Cpa of *C. perfringens* [102], B subunit of the heat-labile toxin (LTB) of *E. coli* [14], and BclA2 of *C. difficile* [107].

It is known that at acidic conditions (pH 3.0–4.0), proteins highly concentrated outside the spore spontaneously and tightly bind to the spore surface, but the molecular mechanism of spore adsorption is still not fully understood. Experiments using the Red Fluorescent Protein (RFP) of the coral *Discosoma* sp. as a model protein revealed that adsorbed molecules cross the outermost surface layer of the *B. subtilis* [108] or *B. megaterium* [109] spores. The outermost spore layer of most/all spore former species is characterized by the presence of cysteine-rich proteins that self-assemble into hexameric complexes, producing a protein lattice permeated by pores (Figure 4) [49,50,51,100]. Such pores mediate the spore permeability to germinants [110] and, in *B. subtilis*, are controlled by the spore surface protein CotG [111].

A model proposed to explain spore adsorption suggests that adsorbed protein infiltrates through the pores present in the outermost layer and localizes between the outermost layer (exosporium or outer coat), and the immediately underneath layer (Figure 4) [100].

It has been reported that small proteins often adsorb more efficiently than big proteins, but this trend is not always followed, suggesting that the permeability of the pores depends on the size of the adsorbed protein, but also on the physico-chemical properties of both the heterologous protein and the spore [100]. The isoelectric point, the electric charge, and the relative hydrophobicity of the molecule to be adsorbed have been suggested as relevant for efficient adsorption [102,112]. The same heterologous proteins are adsorbed with different efficiencies by spores of different species [109] or by different strains of the same species [113], or even by spores of the same strain but grown at different conditions [114], suggesting that the different physicochemical properties of the spore surface influence the efficiency of adsorption.

According to the proposed model, the adsorbed proteins would be mainly localized inside the spore and only in a minimal part exposed on the spore surface [100]. The internal localization of the adsorbed proteins would then explain the tight adhesion and the higher stability and resistance to unfavorable conditions of adsorbed over free proteins [100].

In some cases, the standard spore-adsorption procedure (Figure 4) has been used in a modified version. Heat-inactivated spores of *B. subtilis* have been successfully used to bind influenza H5N1 virions (NIBRG-14; clade 1) [115] or a *Mycobacterium tuberculosis* antigen [116], introducing the concept of using spores as inert displaying bioparticles.

### 4.4. Recombinant vs. Non-Recombinant Spore Display

Both spore display approaches have advantages and disadvantages, and the preference for one or the other system has to be analyzed case-by-case. The recombinant approach has the clear advantage that the antigen does not have to be produced and purified. The recombinant bacterium contains the gene coding for the antigen fused to a spore surface protein; therefore, the sporulating cell produces the antigen to be displayed, reducing the costs and simplifying the production process. On the other end, the non-recombinant system has the obvious advantage of being non-recombinant, thus, it does not raise safety concerns related to the use and environmental release of recombinant spores.

In addition to these, the non-recombinant approach is significantly more efficient than the recombinant system. Isticato et al. [14] compared the efficiency of the two display approaches by using the same antigen, LTB of *E. coli*. An average of 9.6 × 10^−5^ pg per spore of the CotC-LTB fusion protein was displayed by a strain carrying a *cotC::eltB* gene fusion [62], while up to 2.5 × 10^−3^ pg of LTB per spore were adsorbed to wild type *B. subtilis* spores [14]. This roughly 25-fold increase of displayed LTB becomes even larger (up to 100-fold increase) by using mutant spores altered in the spore surface [14]. A significantly increased efficiency of display is particularly relevant for a vaccine delivery system, since it allows reducing either the number of spores/dose or the number of doses needed to deliver a sufficient amount of antigen for the induction of an antigen-specific immune response.

An additional relevant advantage of the non-recombinant system is that multimeric antigens are presented in their mature conformation. LTB of *E. coli* is a pentamer that only in its native form is functional and binds its receptor, the GM1 ganglioside, on the enterocyte surface [117]. When expressed as a fusion protein on *B. subtilis* spores [62] or on the surface of *S. gordonii* [118], LTB is displayed as a monomer, while LTB pentamers are displayed by the non-recombinant system on *B. subtilis* spores [14].

However, the non-recombinant spore display system also has disadvantages: (i) displayed antigens are not on the spore surface and can be presented to the immune cells only after the spore germinates or is destroyed in the animal body; (ii) the molecular mechanisms of spore adsorption are still not fully understood. According to the model of Figure 4, antigens would accumulate within the spore surface layers in a disordered way, thus impairing the construction of precise structures, with epitopes exposed in the most convenient way for interaction with the immune cells.

## 5. Mucosal Immunizations with Recombinant and Non-Recombinant Spores

Spores that do not display any heterologous antigen induce low levels of spore-specific IgG response when they are mucosally (orally or nasally) administered to mice [57]. While they are not recognized by TLR2 and TLR4, the two principal Toll-like receptors sensing live bacteria, and seem unable to interact with B cells, spores stimulate the induction of IFN-γ and other mediators of a cellular response [119]. In addition, orally administered spores have been shown to reduce the susceptibility to enteric pathogens in animal models. D’Arienzo et al. [59] used *Citrobacter rodentium*, a mouse pathogen causing epithelial lesions similar to those caused by human enteropathogenic and enterohemorrhagic strains of *E. coli* [120], to show the protective effects of *B. subtilis* spores. In this infection model, a treatment with 1 × 10^9^ spores one day before the infection with *C. rodentium* drastically decreased colon colonization, prevented the enteropathy by reducing crypt length and epithelial damages, and significantly reduced the mortality rate [59]. In other studies, a single oral inoculum of spores suppressed all signs of *Escherichia coli* O78:K80, *Salmonella enterica* or *Clostridium perfringens* infections in chickens [121,122].

Spores displaying heterologous antigens by the recombinant approach have been shown to induce antigen-specific immune responses in mice orally or nasally immunized. In this context, the best-characterized example is that of spores displaying the C-terminus of the tetanus toxin (TTFC) fused to the spore surface protein CotB of *B. subtilis.* Mice orally immunized with those recombinant spores produced TTFC-specific antibodies (fecal sIgA and serum IgG) with an IgG isotype profile, indicating a prevalence of IgG1 and IgG2b and, therefore, a Th2-type immune response [41]. When challenged with the purified tetanus toxin, the immunized animals were fully protected, demonstrating the potential of spores as a valuable delivery system for mucosal vaccines [41]. The same spores also induced a significant TTFC-specific IgA and IgG response with a prevalence of IgG1 and IgG2b, indicative of a Th2-biased immune response when used to orally prime mice that were then subcutaneously boosted with soluble TTFC (without adjuvant) [123]. When orally administered, the same spores were also shown to induce a cellular immune response in Balb/C mice with spleen and mesenteric lymph nodes (MLN) cell proliferation, as well as the production of IFN-γ, but not of IL-4 and IL-10 in both districts [118]. When the CotB-TTFC chimera was displayed on the surface of spores of a mutant strain of *B. subtilis* unable to germinate, similar levels of cell proliferation and a similar pattern of cytokine induction were observed with respect to those observed with wild-type spores, indicating that the observed antigen-specific cellular immune response was independent from spore germination in the GIT, and was only due to the antigen exposed on the orally ingested spores [124]. The same conclusion was also reached using a different antigen, the MPT64 of *Mycobacterium tuberculosis* [73]. In this case, spores displaying the antigen fused to CotB were heat-inactivated and were still able to induce an immune response reducing the pathogen load in the animal lungs and inducing the secretion of Th1 cytokines [73].

Other examples of antigens displayed on spores as fusion proteins and able to induce a strong immune response when mucosally administered are the LTB of *E. coli* [60], the C-terminus of the alpha toxoid of *Clostridium perfringens* (Cpa) [116], and the BclA_3_ of *C. difficile* [74]. In the LTB and Cpa cases, high levels of antigen-specific IgG and sIgA were induced [62,68], and recombinant spores displaying Cpa fused to CotB protected the orally or nasally immunized mice against a 12 LD_50_ challenge dose of alpha toxin [68]. Also, spore-displayed BclA3 induced antibody production in mice and attenuated some *C. difficile* infection symptoms after a challenge with the pathogen, but was less efficient than the free antigen [74].

More recently, SARS-CoV-2 antigens have been fused to CotB or CotC and recombinant spores used to immunize hamsters [79]. Golden Syrian hamsters were primed intramuscularly with the recombinant Spike protein followed by two intranasal boosts with a mixture (1:1) of recombinant spores expressing either the receptor binding domain (RBD) (CotB-RBD) or the HR1-HR2 fragment (CotC-HR1HR2) of the SARS-CoV-2 Spike protein [REF]. The viral load decreased progressively in the oropharingeal tract and in the lungs of vaccinated animals [79].

The effects of some antigens delivered by spore adsorption have also been characterized. Huang et al. [102] reported that purified Cpa of *C. perfringens* mixed to *B. subtilis* spores induced an immune response indistinguishable from that induced by recombinant spores displaying Cpa fused to CotB [102]. Mice were immunized with either three oral doses of spores mixed with 3.6 μg of Cpa or a single nasal dose of spores mixed with 0.15 μg of Cpa. Protection was obtained in nasally dosed mice to a 6 LD50 dose of toxin, while for oral dosing, only one mouse survived, suggesting that with the non-recombinant approach, the nasal route is preferable to the oral one [102]. In the same study, spores were also mixed with TTFC of *C. tetani* and PA of *B. anthracis* and in all cases, a Th1-biased immune response was observed and the immunized animals were protected against the challenge with the purified toxins [102]. In all cases, heat-inactivated spores appeared equally effective as live spores [102]. This remarkable result was further exploited, showing that killed spores adsorbed to inactivated influenza virions (H5N1; NIBRG-14; clade 1) induce both humoral and cell-mediated immune responses when intra-nasally administered to mice [115]. In a challenge experiment, mice nasally dosed two times with spores adsorbed with 20 ng hemagglutinin of inactivated NIBRG-14 were fully protected against challenge with 20 LD50 of H5N2 virus [115]. Humoral and cellular immune responses were also observed in mice nasally immunized with spores adsorbed with LTB of *E. coli* [14]. Production of fecal and serum sIgA, serum IgG, and IFN-γ by both spleen and MLN cells of mice immunized with spore-adsorbed LTB was observed at levels statistically higher than those observed by immunizing mice with purified LTB, an effect that could be related to an increased antigen uptake by competent immune cells or, alternatively, to a reduced antigen degradation [14]. An increased antigen stability was observed when the spore surface protein BclA2 of *C. difficile* was adsorbed to *B. subtilis* spores [107]. In addition, spores adsorbed with BclA2 showed an increased adherence to human intestinal (Caco-2) cells in vitro, and induced antigen-specific antibody production in nasally immunized mice [107].

Spore-adsorbed TTFC, when nasally administered to mice, was more efficient than the free antigen in inducing fecal sIgA, serum IgG, and the cytokines IL-6 and IL-12 [24]. The efficiency of the nasal vaccination was further improved by oral probiotic treatment with *B. toyonensis* [24]. In this case, both the humoral and the cellular immune responses were enhanced by the probiotic treatment without significantly altering the gut’s microbial composition, pointing to the probiotic treatment as an alternative to the use of adjuvants for mucosal vaccinations [24].

## 6. Future Perspectives

Both recombinant and non-recombinant systems of spore display are potentially efficient strategies to deliver heterologous antigens to mucosal surfaces. Each system has advantages and disadvantages, leaving the preference to one or the other to a case-by-case analysis. An exciting future perspective is the combined use of both systems. Examples are the use of recombinant spores displaying a protein able either to act as an adjuvant [125] or specifically target the spore to a tissue or cell type [126] that can be adsorbed with a different molecule. In this context, *B. subtilis* spores displaying the adjuvant IL-2 or streptavidin as a chimeric fusion were adsorbed with the purified antigen FliD of *C. difficile* [125] or the diterpen paclitaxel, a mitotic inhibitor used in cancer therapy [126]. By this approach, spores displaying IL-2 showed an increased immune response [125], and those displaying streptavidin can bind any biotinylated antibody, potentially targeting spores and adsorbed molecules to any potential target cell therapy [126].

Most of the experiments so far performed to display antigens have been carried out with laboratory collection strains. An additional, intriguing future perspective is to display antigens on spores of probiotic strains of *B. subtilis* or other *Bacillus* strains. Several strains of spore former species are widely used as probiotics for human or animal use [60,61], and the possibility of using spores of probiotic strains to display antigens would allow combining the beneficial probiotic effects to the induction of an antigen-specific immune response.

## Figures and Tables

**Table 1:** Licensed mucosal vaccines for human use ^a^.

Pathogen	Trade Name	Composition	Route, Dose	Immunological Mechanism	Efficacy
Rotavirus	Rotarix; RotaTeq	Live attenuated	Oral, 3 doses	sIgA and systemic neutralizing IgG	Over 70–90%
Poliovirus	Orimune; OPV; Poliomyelitis vaccine	Live attenuated	Oral, 3 doses	sIgA and systemic IgG	Over 90%
*Salmonella typhi*	Vivotif; Ty21A	Live attenuated	Oral, 3–4 doses	sIgA, systemic IgG and CTL responses	Variable, but more than 50%
*Vibrio cholera*	Dukoral; ORC-Vax; Shanchol	Inactivated *V. cholera*	Oral, 2–3 doses	Antibacterial, toxin-specific and LPS-specific IgA	Over 85%
Influenza Virus A	FluMist Quadrivalent^®^	Antigens incorporated into live attenuated, cold adapted influenza vector	Nasal, 1 dose	Mucosal IgA and systemic IgG	Over 90%
Influenza Virus A and B	Fluenz Tetra^®^	Antigens into live attenuated, cold-adapted influenza vector	Nasal, 1 dose	Mucosal IgA, systemic IgG and CTL responses	Variable, but more than 50%

^a^ Data from references [2,7].

**Table 2:** Coat proteins of *B. subtilis* proposed as carriers to display antigens.

Carriers	Antigens	References
CotB	TTFC of *Clostridium tetani*	[40]
	LTB of *Escherichia coli*	[62]
	FliD of *Clostridium difficile*	[63,64]
	PA of *Bacillus anthracis*	[65]
	UreA of *Helicobacter acinonychis*	[66]
	TcdA-TcdB of *Clostridium difficile*	[67]
	Cpa of *Clostridium perfringens*	[68]
	VP28 of White Spot Syndrome Virus	[69,70]
	M2 protein of influenza virus	[71]
	SlpA of *Lactobacillus brevis*	[72]
	InvA of *Yersinia pseudotuberculosis*	[72]
	MPT64 of *Mycobacterium tuberculosis*	[73]
	BclA3 of *Clostridium difficile*	[74]
	VP1 capsid protein of Enterovirus 71	[75]
	HR2P of SARS-CoV-2 spike	[76]
	PCV2 Cap protein of *Porcine circovirus*	[77]
	Vp7 of grass carp reovirus	[78]
	RBD of SARS-CoV-2 spike	[79]
CotC	TTFC of *Clostridium tetani*	[40]
	LTB of *Escherichia coli*	[62]
	FliD of *Clostridium difficile*	[63,64]
	PA of *Bacillus anthracis*	[65]
	UreA of *Helicobacter acinonychis*	[66]
	TcdA-TcdB of *Clostridium difficile*	[67]
	UreB of *Helicobacter pylori*	[80]
	TP22.3 of *Clonorchis sinensis*	[81]
	CsSerpin3 of *Clonorchis sinensis*	[82]
	Pep23 of HIV	[83]
	GST of *Schistosoma japonicum*	[84]
	GP64 of *Bombyx mori*	[85]
	Enolase of *Clonorchis sinensis*	[86]
	Paramyosin of *Clonorchis sinensis*	[87]
	OmpC of *Salmonella serovar Pullorum*	[88]
	VP4 of Grass carp reovirus	[89]
	VP56 of Grass carp reovirus	[90]
	Vp26 of White spot syndrome virus	[69,91]
	Vp7 of grass carp reovirus	[78]
	MCP of Nervous necrosis virus (RGNNV)	[92]
	HR1HR2 of SARS-CoV-2 spike	[79]
	Sip of Streptococcus agalactiae	[93]
CotG	UreA of *Helicobacter acinonychis*	[66]
	FliD of *Clostridium difficile*	[63,64]
CotY	OmpK of *Vibrio vulnificus*	[94]
	RBD of SARS-CoV-2 spike	[95]
CotZ	FliD of *Clostridium difficile*	[63,64]
	UreA of *Helicobacter acinonychis*	[66]
	RBD of SARS-CoV-2 spike	[95]
CgeA	CagA of *Helicobacter pylori*	[96]

## Data Availability

All data are reported in the papers indicated as references.
